# Adult Knowledge About Postoperative Complications of Rhinoplasty in the Western Region of Saudi Arabia

**DOI:** 10.7759/cureus.37183

**Published:** 2023-04-05

**Authors:** Medhat Taha, Hassan Ali A AlZubaidi, Ahmed Ali A Alrezqi, Abdulaziz Mohammed O Alsokani, Abdullah Aqeel A Alrashdi, Abdulwahab Abdulaziz K Alzubaidi, Ali Saleh A Alqarni

**Affiliations:** 1 Department of Anatomy, Umm AlQura University, Al-Qunfudah College of Medicine, Al-Qunfudah, SAU; 2 Department of Medicine and Surgery, Umm AlQura University, Al-Qunfudah College of Medicine, Al-Qunfudah, SAU

**Keywords:** saudi arabia, western region, rhinoplasty, postoperative, complications

## Abstract

Background

Rhinoplasty, a prevalent cosmetic surgical procedure worldwide, is not exempt from associated risks and complications, as with any other surgery. In light of the surging demand for rhinoplasty amongst young adults, it is imperative to acknowledge that the procedure can engender various complications that can be classified as either early or late complications. Examples of early complications include epistaxis and periorbital ecchymosis, while late complications may manifest as enophthalmos or septal perforation. The present study endeavors to gauge the knowledge of rhinoplasty complications among adult residents of the Western region of Saudi Arabia.

Methods

To achieve the research objectives, a cross-sectional study design was implemented, utilizing a self-administered online questionnaire. The study targeted male and female adults aged 18 years and above residing in the Western region of Saudi Arabia. The questionnaire comprised of 14 items, categorized into socio-demographic and rhinoplasty postoperative complications sections, respectively.

Results

The study gathered responses from a total of 968 participants, of which 60.95% fell within the age range of 18-30 years. The majority of participants identified as female (77.89%), and Saudi citizens constituted the vast majority of the respondents (96.28%). Among the participants, 22.62% expressed a desire to undergo rhinoplasty, whereas 77.38% indicated no interest in the procedure. Of those who sought rhinoplasty, the majority favored having the surgery performed by a skilled physician (81.74%). Notably, participants exhibited a relatively high level of awareness regarding the postoperative complications of rhinoplasty, with respiratory issues being the most widely recognized complication (66.63%). Conversely, headache, nausea, and vomiting were the least familiar complications (100%).

Conclusion

The study findings reveal a considerable knowledge gap among adults residing in the Western region of Saudi Arabia concerning rhinoplasty's possible postoperative complications. The results underscore the pressing need to establish comprehensive educational and awareness-raising programs to equip individuals contemplating the procedure with the requisite information to make informed decisions. Future research endeavors could delve into the underlying determinants that drive the desire for rhinoplasty and assess potential interventions geared toward augmenting individuals' comprehension and knowledge of the procedure.

## Introduction

Rhinoplasty, a surgical procedure with cosmetic and functional purposes, is well-known worldwide, especially for its ability to improve obstructed airways [1]. In the United States, rhinoplasty constitutes 15% of all cosmetic surgical procedures, with 2,314,720 procedures performed in 2020 [2]. In Saudi Arabia, rhinoplasty accounted for 60% of all plastic surgeries in 2019, with social media playing a crucial role in its popularity [3]. However, as with any surgical procedure, rhinoplasty carries the risk of complications, ranging from mild discomfort to life-threatening risks such as death [4]. These complications are classified as early or late, with early complications including epistaxis, periorbital ecchymosis, septal hematoma, infection, and skin necrosis, while late complications include scar hypertrophy, septal perforation, and enophthalmos [5]. The complication rate ranges from 4% to 18.8%, with life-threatening complications occurring in 1.7% to 5% of rhinoplasty procedures [6,7]. Previous studies have shown that young adults, especially teenage females, lack proper knowledge about rhinoplasty complications and the procedure itself [8]. Therefore, this study aims to investigate the awareness of rhinoplasty postoperative complications among male and female adults aged 18 years or above living in the western region of Saudi Arabia.

## Materials and methods

In this study, a self-administered online questionnaire was used to collect data from adult residents of the Western Region of Saudi Arabia. The study aimed to investigate participants' awareness of postoperative complications of rhinoplasty. A total of 968 participants were recruited randomly using WhatsApp broadcast messages that included the questionnaires, rationale, and objectives. The inclusion criteria specified both male and female adults aged 18 years or older. Data collection took place between 22 September 2022 and 22 October 2022.

The questionnaire consisted of 14 questions, divided into two sections. The first section aimed to collect demographic information about the participants, including age, gender, relationship status, education level, occupation, region of residence, household income, and parental status. The second section included questions about rhinoplasty reconstructive surgery and its complications to ensure the objectivity and completeness of the study.

Prior to participation, all participants provided informed consent, and data confidentiality was ensured. The questionnaire used in this study was adopted from a previous study that covered the presented topic [8]. The study design was cross-sectional, and the data collected were analyzed using appropriate statistical methods.

Ethical consideration

Ethical approval was granted by the Institutional Review Board (IRB) of Umm Al-Qura University, with approval number: HAPO-02-K-012-2022-11-1288.

Statistical analysis

The analysis was conducted using the Statistical Package for Social Sciences, Version 26.0 (IBM Corp., Armonk, NY) and data were presented as frequencies and percentages of categorical data.

## Results

In this cross-sectional study conducted in the Western Region of Saudi Arabia, a total of 968 respondents completed an online questionnaire. The majority of respondents were young adults, with 60.95% aged between 18 to 30 years. The majority of respondents were female, constituting 77.89% of the sample. The majority of participants were Saudi citizens, comprising 96.28% of the sample. In terms of marital status, 52.38% of participants were single, and 43.08% were married. Education-wise, the majority of respondents were bachelor's degree holders, accounting for 68.70% of the sample, and the majority of participants were employed (44.63%) or students (35.43%). Geographically, most of the participants lived in Makkah (37.81%), followed by Jeddah (27.58%) (Table 1).

**Table 1:** Demographic characteristics of the study participants n= number of participants, (%) percentage

				n		%	
	Age	<18	20		2.07%		
		18 – 30	590		60.95%		
		31 – 40	162		16.74%		
		41 – 50	124		12.81%		
		51 – 60	63		6.51%		
		> 60	9		0.93%		
	Sex	Female	754		77.89%		
		Male	214		22.11%		
	Nationality	Non -Saudi	36		3.72%		
		Saudi	932		96.28%		
	Social Situation	Divorced	32		3.31%		
		married	417		43.08%		
		Single/single	507		52.38%		
		widow	12		1.24%		
	Education Level	Primary > Elementary	10		1.03%		
		Medium > middle School	14		1.45%		
		high school	139		14.36%		
		diploma	42		4.34%		
		Bachelor	665		68.70%		
		Master's	76		7.85%		
		PhD	22		2.27%		
	Occupation	Student	343		35.43%		
		employed	432		44.63%		
		unemployed	156		16.12%		
		retired	37		3.82%		
	Place of residence in the western region	Al -Qunfudah Governorate	81		8.37%		
		Jeddah Province	267		27.58%		
		Madina El Monawara	173		17.87%		
		Makkah	366		37.81%		
		Taif Governorate	81		8.37%		

The study's findings on participants' perception of their nose's appearance indicate that 62.50% of them were satisfied with their nasal shape, while 16.53% expressed dissatisfaction, and 20.97% remained indifferent. Among the study participants, 22.62% expressed a desire to undergo rhinoplasty, while 77.38% stated that they had no intention of undergoing this procedure. Of those who were willing to undergo rhinoplasty, 36.79% did so for fashion trends, while 22.17% did it for cosmetic concerns related to the shape of their noses. The majority of the participants (81.74%) stated that they would only consider rhinoplasty if it was performed by a reputable surgeon. Conversely, among those who did not want to undergo rhinoplasty, 71.35% admired the shape of their noses and did not see any benefit in cosmetic surgery. Table 2 provides more detailed information on these findings.

**Table 2:** Participants’ attitude toward rhinoplasty percentage (%)

	Category	Frequency		%
How do you feel about the appearance of your nose?	Happy	605	62.50%	
	Not happy	160	16.53%	
	I do not care	203	20.97%	
Do you want to do a plastic surgery for your nose?	No	749	77.38%	
	yes	219	22.62%	
Why do you want to do a nose cosmetic? (If you choose yes)	Confident	1	0.47%	
	Cosmetic concerans	47	22.17%	
	Fashion followers	78	36.79%	
	For psychological stability	22	10.38%	
	Medical reasons	21	9.91%	
	pressure from friends or family	14	6.60%	
	To show off	29	13.68%	
What kind of doctors prefer the operation for you? (if you choose yes)	A doctor receives a little and has good morals	12	5.48%	
	A doctor works well	180	81.74%	
	A lot of preoccupation and has many patients	26	11.87%	
	Sure, a doctor has seen the experiences of the patients with him	1	0.46%	
Why do you not want to do a nose cosmetic? (If you choose no)	Fear of side effects	129	17.77%	
	I am satisfied with my nose shape	518	71.35%	
	I do not have enough money	52	7.16%	
	religious reasons	27	3.72%	

In addition, the study examined participants' understanding of potential complications following rhinoplasty. The results indicated that the most commonly recognized complication was breathing difficulties, with 66.63% of participants acknowledging it. Conversely, the least recognized complications were headache, nausea, and vomiting, with only 100% of participants being aware of them. Further details on these results are given in Table 3.

**Table 3:** Familiarity with some of the post operative complications of rhinoplasty percentage (%)

Category	Percent how know	Percent how did not know
Skin discoloration	40.70%	59.30%
Breathing disorders	66.63%	33.37%
Recurrent nosebleed	35.95%	64.05%
Nose Blockage	34.50%	65.50%
Recurrent nasal mucosal irritation	25.41%	74.59%
Headache	0.00%	100%
Recurrent nausea and vomiting	0.00%	100%
Nasal discharge	3.82%	96.18%
Sensitivity to strong adores	29.75%	70.25%
Death	7.33%	92.67%
Need for reoperation	33.78%	66.22%
Dissatisfaction with new nose	48.45%	51.55%
Mismatch of new nose with the rest of the face	36.05%	63.95%

## Discussion

This study aimed to assess the knowledge and awareness of postoperative complications of rhinoplasty among participants in the western region of Saudi Arabia. Rhinoplasty is a surgical procedure that is performed for both functional and cosmetic reasons to improve nasal airway and breathing. The findings suggest that a significant proportion of the participants lacked knowledge about the potential complications associated with Rhinoplasty, with breathing disorders being the most commonly recognized complication, while headache, nausea, and vomiting were the least known. Most participants expressed satisfaction with the appearance of their noses, with only 22.26% expressing a desire to undergo rhinoplasty, primarily influenced by fashion trends.

These findings align with a previous study conducted on female high school students in Iran, where over half of the participants had poor knowledge of rhinoplasty complications. Breathing disorders were the most well-known complication, followed by dissatisfaction with the results, while nasal discharge was the least known complication [8]. The study suggests that false advertising of rhinoplasty on social media by influencers who have undergone the procedure may contribute to the general public's lack of awareness of rhinoplasty complications [9,10]. A study conducted by Obied et al. found that most participants believed that rhinoplasty is a safe procedure, which supports the hypothesis that the public is not aware of the potential complications associated with the procedure [11]. Another study by Alghamdi et al. reported that nearly 74% of the participants believed that rhinoplasty is a safe surgical procedure, highlighting the impact of social media advertising on public perception [12].

The selection of a qualified and experienced surgeon can play a significant role in increasing awareness of rhinoplasty complications and avoiding them [13]. The majority of participants in this study preferred a surgeon with a high success rate, which is consistent with the findings of a previous study by Mianroodi et al. [8].

Recommendations and limitations

The present study represents a novel investigation in the Kingdom of Saudi Arabia, focusing on the level of awareness surrounding post-operative complications associated with rhinoplasty. Our results emphasize the crucial need to increase public awareness regarding the appropriate indications for rhinoplasty and disseminate evidence-based information about the procedure through social media channels.

Nonetheless, we recognize that there are several limitations to our study that require attention. Firstly, our study sample was limited to a specific social demographic group, potentially limiting the generalizability of our findings to other populations. Secondly, our study was confined to particular regions of Saudi Arabia and did not encompass other provinces, thus restricting access to individuals who underwent rhinoplasty in other regions.

## Conclusions

An online survey involving 968 participants was conducted to assess the level of knowledge and awareness regarding postoperative complications associated with rhinoplasty in the western region of Saudi Arabia. The survey indicated that although breathing disorders were recognized as the most commonly known complication, there was a lack of knowledge regarding potential complications such as headache, nausea, and vomiting. Additionally, the survey revealed that many participants who expressed a desire to undergo rhinoplasty were influenced by fashion trends and cosmetic concerns about the appearance of their noses and preferred surgeons who demonstrated proficiency in the field.

The results of the study reveal a notable dearth of knowledge among the surveyed individuals regarding complications arising from rhinoplasty in the western region of Saudi Arabia. The findings emphasize the importance of public education about the potential risks associated with the procedure and the significance of selecting a skilled and qualified surgeon to reduce the incidence of complications.
